# Anesthetic Management of Patients With Advanced Carotid Artery Disease Undergoing Ophthalmic Surgery: Implications for Cerebral Perfusion and Visual Outcomes

**DOI:** 10.7759/cureus.107481

**Published:** 2026-04-21

**Authors:** Saul J Prado Fonseca, Fabiana M Araya Padilla, Nicole Rodríguez, Natalia Ruiz Rojas, Jose Ignacio Chacon Soto, Gabriel Anchía Jiménez

**Affiliations:** 1 Emergency Medicine, Hospital Monseñor Sanabria Martínez, Puntarenas, CRI; 2 General Medicine, Caja Costarricense de Seguro Social (CCSS), Alajuela, CRI; 3 General Medicine, Hospital Monsenor Sanabria Martínez, Puntarenas, CRI; 4 Emergency Medicine, Hospital Monsenor Sanabria Martínez, Puntarenas, CRI; 5 General Medicine, Universidad de Costa Rica, Alajuela, CRI

**Keywords:** anesthetic management, carotid artery disease, cerebral perfusion, hemodynamic stability, ophthalmic surgery, perioperative risk

## Abstract

Carotid artery disease represents a significant clinical challenge in patients undergoing ophthalmic surgery due to its direct impact on cerebral and ocular perfusion. Its prevalence increases with age and is strongly associated with cardiovascular risk factors such as hypertension, diabetes mellitus, dyslipidemia, and smoking. These patients often present with impaired cerebral autoregulation, making cerebral blood flow highly dependent on systemic blood pressure. Consequently, even minor hemodynamic fluctuations during the perioperative period may precipitate ischemic complications.

The pathophysiology involves both hypoperfusion and embolic mechanisms. Reduced cerebral blood flow, compounded by limited collateral circulation through the Circle of Willis, increases the risk of neurological injury. Simultaneously, embolization of atherosclerotic plaques may lead to acute cerebral or ocular events, including retinal artery occlusion and ischemic optic neuropathy. These risks highlight the importance of maintaining stable systemic and ocular perfusion throughout the surgical process.

Preoperative evaluation requires a comprehensive neurological, cardiovascular, and imaging assessment to stratify risk and guide management. Intraoperatively, the primary objective is to preserve cerebral perfusion by maintaining stable mean arterial pressure and avoiding both hypotension and excessive hypertension. This is achieved through careful titration of anesthetic agents, appropriate fluid therapy, and the use of vasopressors when necessary.

The choice of anesthetic technique should prioritize hemodynamic stability, with regional anesthesia and monitored sedation often preferred. Pharmacological management, including the selective continuation or withholding of medications, further contributes to optimizing outcomes. Overall, a tailored, multidisciplinary approach is essential to minimize perioperative complications and ensure patient safety.

## Introduction and background

Carotid artery disease is of major clinical relevance in the setting of ophthalmic surgery, particularly among older adults undergoing procedures such as cataract and glaucoma surgery. In this population, carotid disease is associated with an increased risk of perioperative cerebral hypoperfusion and embolic events, both of which may lead to serious and potentially irreversible complications, including stroke, permanent neurological deficits, and visual impairment. In this context, a thorough understanding of the underlying pathophysiological mechanisms and associated risk factors is essential for safe and effective anesthetic management [1].

Both cerebral and ocular perfusion are central to the perioperative evaluation of these patients. The ophthalmic artery arises directly from the internal carotid artery, creating a close anatomical and functional relationship in which alterations in carotid blood flow can directly compromise ocular circulation. As a result, impaired perfusion may contribute to ocular ischemic syndrome and other vision-threatening complications, underscoring the need to preserve adequate blood flow throughout the surgical period [1].

Within this framework, anesthetic management plays a pivotal role in reducing perioperative risk. Monitoring cerebral autoregulation through cerebral oximetry has been proposed as a useful strategy to guide intraoperative hemodynamic management, support adequate cerebral perfusion, and potentially reduce the risk of ischemic brain injury, which in severe cases may result in irreversible neurological complications [2]. In addition, near-infrared spectroscopy has been used to detect cerebral ischemia, particularly in carotid endarterectomy; however, its sensitivity remains limited. For this reason, it should be interpreted cautiously and used in combination with other neuromonitoring modalities rather than as a standalone tool, in order to strengthen intraoperative neuroprotective strategies [3]. From a pharmacological perspective, the co-administration of dexmedetomidine with total intravenous anesthesia has been shown to reduce propofol requirements for burst suppression, thereby improving hemodynamic stability and decreasing the need for vasopressors [4].

At the same time, ocular complications related to anesthesia deserve particular attention. These may range from relatively minor conditions, such as corneal abrasions, to severe and irreversible outcomes, including permanent vision loss. Accordingly, a high level of clinical suspicion is essential for prompt recognition and timely management [5]. Retrobulbar anesthesia, in particular, has been shown to decrease intraocular pressure and ocular pulse amplitude, effects that may be detrimental during the intraoperative period, especially in patients with pre-existing retinal or optic nerve damage. In such cases, its use should be approached with caution, and alternative anesthetic techniques should be considered when appropriate [6].

Anesthetic management must therefore be individualized according to the specific clinical profile of each patient. Factors such as age, diabetes mellitus, and the severity of carotid stenosis significantly influence the risk of both cerebral and ocular complications, and careful consideration of these variables may improve perioperative outcomes [7, 8]. This is especially relevant in elderly patients, particularly those older than 80 years, who may exhibit distinct risk profiles and outcomes related to carotid disease and its management. A personalized approach based on overall health status, vascular risk burden, and functional capacity is therefore essential [9].

The objective of this review is to provide a comprehensive and clinically integrated analysis of anesthetic management in patients with advanced carotid artery disease undergoing ophthalmic surgery, with particular emphasis on strategies aimed at preserving cerebral and ocular perfusion and minimizing neurological and visual complications.

## Review

Methodology

This manuscript was developed as a structured narrative review aimed at providing an updated and clinically integrated analysis of anesthetic management in patients with advanced carotid artery disease undergoing ophthalmic surgery, with particular emphasis on the implications for cerebral perfusion and visual outcomes. The review was conducted in accordance with the SANRA [Scale for the Assessment of Narrative Review Articles] framework and followed a predefined methodological protocol established prior to literature screening. Given the clinical heterogeneity of this population, the variability in carotid disease severity, the diversity of ophthalmic procedures, and differences in anesthetic techniques and perioperative monitoring strategies, a narrative interpretative synthesis was selected over quantitative pooling to integrate vascular, neurologic, ophthalmologic, and anesthetic considerations into a coherent and clinically applicable framework. Special attention was given to perioperative hemodynamic management, preservation of cerebral and ocular perfusion, anesthetic technique selection, monitoring modalities, and the prevention of neurologic and visual complications. The objective was to provide a structured synthesis capable of supporting multidisciplinary perioperative decision-making in high-risk patients undergoing ophthalmic surgery [10].

A comprehensive literature search was conducted in PubMed, Scopus, and Web of Science, including peer-reviewed articles published in English or Spanish between January 2020 and December 2025. The final search was performed in March 2026. This timeframe was selected to capture contemporary advances in perioperative hemodynamic monitoring, cerebral oximetry, ophthalmic anesthesia techniques, carotid-related cerebrovascular risk assessment, and strategies aimed at preserving cerebral and ocular perfusion during surgery. Foundational studies were incorporated when necessary to contextualize relevant pathophysiological mechanisms, vascular anatomy, or historical principles of anesthetic and ophthalmic management. The search strategy combined MeSH and free-text terms using Boolean operators related to carotid artery disease, carotid stenosis, ophthalmic surgery, ocular surgery, cataract surgery, glaucoma surgery, regional anesthesia, retrobulbar anesthesia, peribulbar anesthesia, monitored anesthesia care, general anesthesia, cerebral perfusion, ocular perfusion, cerebral oximetry, near-infrared spectroscopy, visual outcomes, and perioperative stroke. Searches were conducted in titles and abstracts as well as indexed subject headings to maximize sensitivity.

The initial search yielded 208 records. After removal of duplicates, 153 articles remained for title and abstract screening. Of these, 92 underwent full-text evaluation, and 46 studies were included in the final synthesis. Selection was performed independently by two authors, with disagreements resolved through discussion and consensus. Exclusion criteria comprised non-peer-reviewed publications, isolated case reports, editorials without substantive clinical data, purely technical descriptions without perioperative or outcome relevance, redundant datasets, and studies not directly addressing anesthetic implications, perfusion-related considerations, neurologic risk, or visual outcomes in patients with carotid artery disease undergoing ophthalmic surgery.

Eligible studies included randomized controlled trials, observational cohorts, systematic reviews, meta-analyses, expert consensus statements, and contemporary international guidelines from anesthesiology, ophthalmology, vascular surgery, and neurology societies. Priority was assigned to multicenter investigations, studies with clearly defined carotid disease severity, and research evaluating perioperative hemodynamic parameters, cerebral and ocular perfusion, anesthetic strategies, neurologic outcomes, visual outcomes, and treatment-related complications. Extracted variables included study design, patient characteristics, degree of carotid stenosis, type of ophthalmic procedure, anesthetic technique, intraoperative monitoring modality, hemodynamic management strategy, neurologic complications, visual outcomes, and reported perioperative adverse events. Methodological quality and internal validity were assessed narratively, considering risk of bias, sample size, follow-up duration, consistency in the definition of carotid disease and ophthalmic outcomes, and reproducibility of reported findings. In cases of conflicting evidence, greater interpretative weight was assigned to higher-level evidence and guideline-supported recommendations.

Reference lists of included studies were manually screened to identify additional relevant publications. Given its narrative design, this review is subject to potential selection bias and does not provide pooled quantitative estimates. Artificial intelligence-based tools were used exclusively to assist in literature organization and structural coherence, whereas critical appraisal, synthesis, and final interpretation were conducted independently by the authors to preserve methodological rigor.

Epidemiology and patient risk profile

Carotid artery stenosis is highly prevalent in the elderly population, with a marked increase in individuals over 75 years of age, in whom cardiovascular disease remains a leading cause of death and disability. In addition to age-related trends, the prevalence of carotid artery disease varies across underrepresented populations, where factors such as income and race influence both the likelihood of diagnosis and access to treatment, thereby contributing to disparities in clinical outcomes [11].

In this context, several well-established risk factors play a central role in the development and progression of carotid artery disease. Hypertension, diabetes mellitus, dyslipidemia, and smoking constitute the primary contributors to atherosclerotic burden within the carotid circulation. Furthermore, patients with concomitant coronary artery disease and peripheral vascular disease exhibit an increased risk of carotid stenosis progression, reflecting the systemic nature of atherosclerosis [11, 12]. Among these factors, diabetes mellitus has been particularly associated with increased perioperative risk during carotid interventions, further complicating clinical management in affected individuals [13].

The clinical presentation of carotid artery stenosis also significantly influences patient outcomes, particularly when comparing symptomatic and asymptomatic populations. Symptomatic patients, defined by the presence of transient ischemic attacks or prior cerebrovascular events, demonstrate a higher risk of adverse outcomes relative to asymptomatic individuals. Conversely, asymptomatic patients undergoing carotid endarterectomy have been shown to achieve better long-term survival outcomes when compared to their symptomatic counterparts, highlighting the prognostic importance of clinical presentation [14].

Beyond individual risk factors and symptomatology, the surgical setting and overall patient condition further contribute to perioperative risk. The environment in which ophthalmic surgery is performed, whether ambulatory or hospital-based, may influence cumulative risk, with hospital settings potentially allowing for more comprehensive monitoring and management of high-risk patients. Additionally, patient frailty represents a critical determinant of surgical outcomes, as frail individuals experience higher rates of postoperative complications, including stroke and myocardial infarction, underscoring the importance of comprehensive preoperative assessment and risk stratification [15].

Pathophysiology of cerebral and ocular hypoperfusion

Severe carotid artery stenosis has a direct impact on cerebral hemodynamics, as it can significantly reduce cerebral blood flow and predispose patients to ischemic events. This reduction is further exacerbated by impairment in dynamic cerebral autoregulation, a key mechanism responsible for maintaining stable cerebral perfusion despite fluctuations in systemic blood pressure [15, 16]. When this autoregulatory capacity is compromised, cerebral perfusion becomes increasingly dependent on systemic blood pressure, thereby elevating the risk of ischemic injury during episodes of hypotension or hemodynamic instability [1].

In this context, the Circle of Willis plays a crucial compensatory role in maintaining adequate cerebral perfusion, particularly in cases of unilateral carotid artery stenosis. Through collateral circulation, it can partially offset reductions in blood flow; however, its effectiveness is highly dependent on anatomical integrity. Variations in its structure, as well as the presence of atherosclerotic changes within the circle itself, may significantly limit its compensatory capacity, resulting in insufficient cerebral perfusion. These anatomical variants can also impair interhemispheric blood flow, thereby increasing the risk of ischemic complications, particularly during procedures such as carotid endarterectomy [17, 18].

The consequences of reduced cerebral perfusion may manifest differently depending on the temporal pattern of hypoperfusion. Chronic hypoperfusion is associated with the development of silent ischemic lesions and progressive cognitive decline, especially in territories supplied by the posterior cerebral artery [19]. In contrast, acute hypoperfusion events, such as those occurring during surgical procedures, may exacerbate pre-existing cerebral ischemia and lead to overt neurological deficits. This distinction underscores the importance of maintaining stable hemodynamic conditions in the perioperative setting [15, 16].

In addition to hypoperfusion, embolic phenomena represent another critical mechanism linking carotid artery disease to cerebral and ocular complications. The embolization of atherosclerotic plaques can result in acute ischemic events affecting both the cerebral and ocular circulations, contributing to conditions such as retinal artery occlusion and ischemic optic neuropathy [20]. Ocular perfusion, in particular, is highly dependent on the balance between systemic blood pressure and intraocular pressure. Any reduction in perfusion pressure may compromise blood flow to the optic nerve and retina, leading to ischemic injury [21, 22].

As a result of these pathophysiological mechanisms, ocular complications such as ischemic optic neuropathy and retinal vascular occlusions may develop, often resulting in significant visual impairment. The risk of these complications is further heightened in patients with compromised systemic and ocular hemodynamics, reinforcing the need for careful perioperative management aimed at preserving both cerebral and ocular perfusion [21, 22].

Preoperative assessment and risk stratification

Preoperative evaluation in patients with carotid artery disease requires a comprehensive assessment of both neurological and medical history in order to identify factors associated with increased cerebrovascular risk. In this context, patients should be carefully evaluated for neurological symptoms such as focal deficits, transient ischemic attacks, or syncope, as these manifestations may indicate underlying cerebrovascular pathology [23]. Additionally, a documented history of prior stroke or carotid revascularization procedures is of particular importance, as these factors significantly influence perioperative risk and must be incorporated into clinical decision-making [24].

This initial evaluation is complemented by a focused physical examination, which provides essential clinical information regarding the severity and implications of carotid disease. The presence of carotid bruits may suggest significant carotid artery stenosis and should prompt further diagnostic investigation [23]. At the same time, establishing a clear baseline neurological status is critical, as it allows for the detection of any perioperative neurological changes and facilitates early identification of complications [24].

Following clinical assessment, diagnostic studies play a central role in confirming the extent of vascular involvement and guiding risk stratification. Carotid Doppler ultrasound, as a non-invasive modality, is widely used to assess the degree of stenosis and to categorize disease severity as mild, moderate, or severe, thereby contributing to risk stratification. In selected cases, advanced imaging techniques such as computed tomography angiography or magnetic resonance angiography may be employed to evaluate the Circle of Willis and collateral circulation pathways, which are essential for determining the patient’s cerebrovascular reserve [18, 25].

In parallel, a global cardiovascular assessment is necessary to determine overall perioperative risk, given the close association between carotid artery disease and systemic atherosclerosis. This evaluation includes the assessment of the risk of myocardial infarction and other cardiovascular events, which may significantly impact perioperative outcomes [23]. Based on the integration of clinical, imaging, and cardiovascular findings, patients are classified according to the severity of stenosis and the presence or absence of symptoms, which in turn guides the urgency and type of intervention [26].

Subsequently, preoperative optimization focuses on minimizing perioperative risk through targeted medical management. Adequate control of blood pressure is essential to reduce the likelihood of perioperative stroke and to maintain stable cerebral perfusion. In addition, pharmacological management may require adjustments in antiplatelet and anticoagulant therapy in order to balance the competing risks of thrombosis and bleeding. Effective perioperative planning relies on interdisciplinary collaboration among anesthesiologists, ophthalmologists, and neurologists, ensuring that management strategies are tailored to the individual patient’s risk profile and clinical condition [20, 23].

Anesthetic techniques for ophthalmic surgery

Local anesthesia techniques represent a cornerstone in the anesthetic management of patients with carotid artery disease undergoing ophthalmic surgery, as they allow for adequate surgical conditions while minimizing systemic hemodynamic disturbances. Among these, the peribulbar block is associated with a lower risk of globe perforation compared to retrobulbar techniques and provides a more diffuse anesthetic effect. The use of adjuvants such as dexmedetomidine has been shown to enhance both the duration and quality of the block, thereby improving its effectiveness and making it a viable option for ophthalmic procedures [27, 28]. In contrast, the retrobulbar block offers deeper anesthesia and is effective in achieving globe akinesia; however, it carries a higher risk of vascular complications and may not be preferred in patients with significant carotid artery disease due to its potential hemodynamic effects [29].

Alternatively, the sub-Tenon block has emerged as a well-tolerated technique with a lower systemic risk profile and is associated with reduced postoperative pain when compared to peribulbar anesthesia. Its safety profile makes it a suitable option for patients with advanced carotid artery disease undergoing ophthalmic surgery, particularly when minimizing systemic impact is a priority [30, 31].

In addition to regional techniques, monitored sedation plays an important role in this setting. One of its main advantages lies in its minimal impact on hemodynamic stability and its facilitation of rapid postoperative recovery, which is particularly beneficial in patients who may not tolerate significant fluctuations in blood pressure or cardiac output. However, despite these advantages, monitored sedation is not without risk, as it may lead to respiratory depression and hypotension, thereby requiring continuous and careful intraoperative monitoring [32].

General anesthesia, on the other hand, is typically reserved for more complex surgical procedures or for patients who are unable to cooperate. While it provides complete immobility and effective analgesia, it is associated with greater hemodynamic instability, which may be particularly problematic in patients with carotid artery disease. Consequently, its use in this population necessitates meticulous management to avoid exacerbating fluctuations in systemic blood pressure and compromising cerebral perfusion [32].

When comparing these techniques, maintaining hemodynamic stability remains the primary objective, especially in patients with significant carotid stenosis. In this regard, sub-Tenon and peribulbar blocks, particularly when combined with appropriate adjuvants, offer a balance between anesthetic efficacy and safety [27, 30]. Ultimately, the selection of the anesthetic approach should be individualized, taking into account patient-specific factors such as the degree of carotid stenosis, baseline neurological status, and the specific requirements of the surgical procedure [33].

Intraoperative hemodynamic management

Intraoperative hemodynamic management in patients with advanced carotid artery disease undergoing ophthalmic surgery is primarily focused on maintaining adequate cerebral perfusion, which depends on the preservation of stable systemic hemodynamics throughout the procedure. In this context, blood pressure control represents a central component of management, with mean arterial pressure serving as a key parameter. It is recommended to maintain intraoperative mean arterial pressure at or above 60 mm Hg in at-risk patients in order to prevent hypotension and ensure sufficient cerebral perfusion [34]. In settings such as carotid endarterectomy, mean arterial pressure should be titrated according to cerebral oximetry indices, as these reflect the adequacy of cerebral perfusion and allow for more individualized hemodynamic targets [1].

At the same time, careful management of intraoperative hypertension is essential, as excessive elevations in blood pressure may increase the risk of embolization while abrupt reductions may precipitate hypoperfusion. In this regard, near-infrared spectroscopy-guided blood pressure management has been shown to reduce vasopressor requirements and perioperative complications by facilitating more precise hemodynamic control. Therefore, maintaining blood pressure within an optimal range is critical to avoid both hypoperfusion and embolic risk [35].

The prevention of hypotension is another key objective, particularly given the dependence of cerebral perfusion on systemic pressure in patients with impaired autoregulation. Avoiding the overdosage of anesthetic agents is essential in this regard, as excessive sedation may lead to significant decreases in blood pressure. The co-administration of dexmedetomidine with total intravenous anesthesia has been shown to reduce the required dose of propofol, thereby minimizing the risk of hypotension and contributing to improved hemodynamic stability [4]. In addition, the titrated use of vasopressors such as phenylephrine and norepinephrine is recommended to support mean arterial pressure and prevent hypotensive episodes [34, 36].

Fluid therapy also plays a critical role in maintaining hemodynamic stability, as hypovolemia can directly contribute to hypotension and compromised cerebral perfusion. For this reason, fluid administration should be carefully managed, and individualized hemodynamic strategies, including computer-assisted approaches, have demonstrated the ability to reduce intraoperative hypotension by optimizing the balance between fluid therapy and vasopressor use [36].

Monitoring strategies are essential to guide intraoperative management and detect early hemodynamic instability. Continuous arterial pressure monitoring is recommended in high-risk patients, as it allows for more precise detection and correction of hypotensive episodes compared to intermittent measurements [34]. Additionally, the use of near-infrared spectroscopy to monitor cerebral oxygenation provides valuable information for tailoring blood pressure targets and ensuring adequate cerebral perfusion, particularly in procedures involving carotid pathology [35, 37].

Maintaining a stable heart rate and cardiac output is crucial to ensure adequate tissue perfusion and prevent secondary hemodynamic compromise. Avoiding severe bradycardia and fluctuations in cardiac output requires careful titration of anesthetic agents and vasoactive medications, thereby contributing to overall hemodynamic stability during the intraoperative period [38].

Monitoring of cerebral and ocular perfusion

Near-infrared spectroscopy represents a non-invasive modality that allows the measurement of regional cerebral oxygen saturation and has been increasingly utilized for the early detection of cerebral desaturation. Its application has demonstrated effectiveness in cardiac surgery settings, particularly in reducing neurological complications; however, its impact in non-cardiac procedures remains less clearly defined [39]. In the context of carotid endarterectomy, near-infrared spectroscopy has shown high specificity but limited sensitivity for the detection of cerebral ischemia, indicating that it should not be used as a standalone monitoring tool but rather in combination with other modalities to improve diagnostic accuracy [3]. Furthermore, evidence from randomized controlled trials has not demonstrated a significant reduction in cerebral ischemic lesions when near-infrared spectroscopy is used to guide optimization of cerebral oxygenation during these procedures, highlighting ongoing uncertainty regarding its clinical impact [37].

In parallel, transcranial Doppler serves as an important tool for evaluating blood flow within cerebral arteries and for detecting dynamic changes in cerebral hemodynamics. When combined with transfer function analysis, it enables the assessment of dynamic cerebral autoregulation, which plays a crucial role in predicting the development of silent ischemic lesions following carotid artery stenting. This technique is particularly valuable for identifying impaired cerebral autoregulation, a condition that has been recognized as a significant predictor of ischemic events and adverse neurological outcomes [15].

Continuous monitoring of arterial blood pressure constitutes another fundamental component of intraoperative management, as it allows real-time adjustment of perfusion parameters to maintain both cerebral and ocular perfusion. Adequate blood pressure control, particularly when guided by indices derived from cerebral oximetry, may contribute to a reduction in postoperative cerebral ischemia, although current evidence remains limited and continues to evolve. In addition to cerebral monitoring, ocular perfusion can be indirectly assessed by considering the relationship between systemic blood pressure and intraocular pressure. Although direct monitoring of ocular perfusion is not routinely feasible, maintaining stable systemic hemodynamics remains essential to support adequate perfusion of the optic nerve and retina [1].

The integration of multiple monitoring modalities, including near-infrared spectroscopy, transcranial Doppler, and continuous blood pressure monitoring, provides a more comprehensive assessment of cerebral and ocular perfusion. This combined approach allows for a more individualized intraoperative strategy, adapting management to the specific physiological conditions of each patient and potentially improving clinical outcomes. Nevertheless, several limitations must be considered, including the restricted availability of advanced monitoring technologies and variability in their interpretation among clinicians [3, 39]. Additionally, near-infrared spectroscopy is affected by inherent technical limitations, such as reduced spatial resolution and susceptibility to extracerebral signal contamination, which may compromise its accuracy in certain clinical scenarios [40].

Pharmacological and perioperative considerations

Pharmacological management in patients with advanced carotid artery disease undergoing ophthalmic surgery requires careful consideration of hemodynamic stability and the balance between thrombotic and hemorrhagic risks. In this context, the continuation of antihypertensive therapy is generally recommended in order to avoid rebound hypertension; however, specific classes such as angiotensin-converting enzyme inhibitors and angiotensin receptor blockers may be withheld preoperatively due to their association with an increased risk of intraoperative hypotension. Evidence suggests that withholding these agents can reduce the incidence of intraoperative hypotension without increasing postoperative complications, supporting a selective and individualized approach to their perioperative management [41, 42].

With regard to antithrombotic therapy, antiplatelet agents are typically continued in ophthalmic surgery, particularly in patients with carotid artery disease, in order to reduce the risk of thromboembolic events. In contrast, the management of anticoagulants requires a more individualized assessment, balancing the risk of thrombosis against the potential for perioperative bleeding. Available evidence, including data from neurosurgical settings, suggests that early resumption of antithrombotic therapy does not significantly increase hemorrhagic risk, which may inform perioperative decision-making in selected patients [43].

Sedative selection is another critical aspect of anesthetic management, particularly given its impact on hemodynamic stability. Propofol remains widely used but is associated with a risk of hypotension, which may be detrimental in patients with impaired cerebral autoregulation. Alternatives such as ciprofol have demonstrated improved hemodynamic stability and reduced vasopressor requirements in carotid surgery settings. Midazolam, which exerts a lesser effect on systemic hemodynamics, may also be considered; however, its potential for cumulative effects should be taken into account [44].

Analgesic strategies should aim to minimize sympathetic activation, as excessive autonomic responses may contribute to hemodynamic instability. Therefore, agents that do not interfere with cerebral autoregulation are preferred in this patient population [45]. Similarly, the selection of anesthetic agents should prioritize the preservation of cerebral autoregulatory mechanisms. In this regard, ciprofol has shown advantages over propofol in maintaining mean arterial pressure and reducing postoperative complications [44]. Additionally, dexmedetomidine, when used in combination with propofol, has been shown to reduce the required propofol dose and improve overall hemodynamic stability, making it a valuable adjunct in carotid-related procedures [4].

Pharmacological interactions must be considered as part of the overall anesthetic plan. Magnesium may be utilized as an antihypertensive agent with additional antiarrhythmic properties; however, its use has been associated with an increased need for postoperative vasopressors [46]. Conversely, dexmedetomidine may reduce vasopressor requirements due to its propofol-sparing effect and peripheral alpha-agonist activity, further supporting its role in optimizing intraoperative hemodynamic control [4].

## Conclusions

The perioperative management of patients with advanced carotid artery disease undergoing ophthalmic surgery requires a highly individualized and multidisciplinary approach, as cerebral and ocular perfusion is critically dependent on maintaining stable hemodynamics. Factors such as impaired cerebral autoregulation, embolic risk, and variability in collateral circulation significantly increase susceptibility to ischemic complications, making careful preoperative assessment, tailored anesthetic technique selection, and precise intraoperative monitoring essential to optimize neurological and visual outcomes.

Anesthetic strategies that prioritize hemodynamic stability, appropriate pharmacological management, and the integration of multimodal monitoring techniques - such as cerebral oximetry, transcranial Doppler, and continuous blood pressure monitoring - are fundamental in reducing perioperative complications. However, limitations in monitoring sensitivity and variability in patient response underscore the importance of combining clinical judgment with individualized perioperative planning to effectively mitigate the risks of cerebral hypoperfusion, embolization, and visual impairment.
